# The Pork Food Chain as a Route of Transmission of Antimicrobial Resistant *Escherichia coli*: A Farm-to-Fork Perspective

**DOI:** 10.3390/antibiotics12020376

**Published:** 2023-02-11

**Authors:** Martina Rega, Laura Andriani, Antonio Poeta, Silvia Bonardi, Mauro Conter, Cristina Bacci

**Affiliations:** 1 Food Hygiene and Inspection Unit, Veterinary Science Department, University of Parma, Strada del Taglio, 10, 43126 Parma, Italy; 2 Azienda Unità Sanitaria Locale (AUSL) Sede Reggio Emilia, Via Amendola 2, 42122 Reggio Emilia, Italy

**Keywords:** AMR, pork, *E. coli*, food chain, farm-to-fork, one health

## Abstract

Antimicrobial resistance (AMR) is a public health risk that needs to be faced from a One Health perspective that includes humans, animals, and environmental health. The food production chain has been identified as a possible route of transmission of AMR bacteria to humans. The most critical phenomenon is related to Critically Important Antimicrobial (CIA) resistance. β-lactams antibiotics (cephalosporin of 3rd, 4th generation, carbapenem, monobactams, and penicillins), quinolones, aminoglycosides, polymyxin, and glycylcyclines were the CIAs chosen in this study. Samples derived from all the stages of the pork food production chain were collected, including pig feces, carcasses, and pork food products (fresh meat, fermented, and seasoned). *Escherichia coli* were isolated, and AMR and MDR profiles were evaluated. Enterobacterial Repetitive Intragenic Consensus (ERIC-PCR) was used to evaluate phylogenetic similarities. Data showed that 50% of phenotypical AMR observed in the entire pork food chain were related phylogenetically. The contamination of fresh meat, in half of the cases, was not directly related to contamination from feces or carcasses. Despite this, some similarities were found between feces and carcasses. In group analysis, phylogenetic similarities were detected in a 3/36 cluster (8.3%). Nevertheless, further studies are needed to improve consumer risk communication and access to clear and reliable information and health concerns on food labels.

## 1. Introduction

Antimicrobial resistance (AMR) is one of the most significant public health risks that the world currently faces [1]. Decreased sensitivity of microorganisms to commonly used drugs increasingly affects human, animal, and environmental health [2]. The indiscriminate use and misuse of antimicrobials and unprescribed animal feed additives to improve production can have a large impact on the emergence and dissemination of AMR in the food animal industry [3]. In fact, many studies suggest that antimicrobials used in food-producing animals may be directly related to the increase in antimicrobial resistance [4,5,6].

Actively monitoring the emergence and the spread of AMR is essential, as is understanding the underlying molecular mechanisms involved, to develop novel strategies to combat AMR [7,8]. The possible consequences of not safeguarding antimicrobial use have been reported as having a negative impact on health, the economy, and the industry sector [2].

The monitoring of AMR in the food chain was established in 2003 by the Directive on the monitoring of zoonoses and zoonotic agents [9]. The European Commission, under scientifical guidance from the European Food Safety Authority (EFSA), has provided valuable information and decisions for the establishment of targeted animal populations or food categories and bacterial hazards [10].

*Escherichia coli (E. coli)* is a gram-negative bacterium that, although often considered a commensal, can be pathogenic and present both a clinical and an epidemiological challenge [11]. It is commonly found in the intestinal tract of humans and warm-blooded animals, and its ability to acquire multidrug resistance makes it a good bioindicator for monitoring AMR [12].

Pork and poultry meat have been identified as a source of transfer of antimicrobial-resistant microorganisms to humans, and a recent study estimated the probability of 1.5% exposure to resistant *E. coli* through meat consumption [13]. Food of animal origin may be contaminated with antimicrobial-resistant bacteria in many ways. Contamination easily occurs during slaughter and food processing. Antimicrobial resistance can also be transferred from one microorganism to another (conjugation) during food manipulation, thus allowing the spread of AMR caused by different food matrices [13]. In fact, the increasing demand for raw meat carries the risk of ingesting live, non-stressed antimicrobial-resistant bacterial cells [14]. Different food processing and preservation techniques can extend the products’ shelf life thanks to inhibiting their bacterial flora. Despite this, stressed or sub-lethally damaged bacteria cells can be a source of free bacterial DNA, including the eventual presence of antimicrobial resistance genes [13,15].

The World Health Organization (WHO) has ranked antimicrobial agents as Critically Important Antimicrobials (CIAs) to human health, defining risk management related to the use of these drugs [16]. The antimicrobial classes analyzed in the present study belong to CIAs and are β-lactams antibiotics (cephalosporin of 3rd, 4th generation, carbapenem, monobactams, and penicillins), quinolones, aminoglycosides, polymiyxin, and glycylcyclines and were chosen because of their classification as high or highest priority molecules. This classification is based on the availability of alternative antimicrobial therapies to treat serious bacterial infections, particularly in bacteria that may be transmitted to humans by a non-human source and bacteria that can acquire resistance genes from non-human sources [17]. From 2010 to 2020, veterinary antimicrobials sales decreased by 30%, also due to a national action plan introduced in 2017 to reduce the use of CIAs by 10% in three years [18,19]. Despite this, Italy is one of the EU countries with the highest veterinary antimicrobials sales, after Germany, Spain, the United Kingdom, France, and Poland [20].

The present study focuses on the pork food chain from a farm-to-fork perspective. Pigs’ feces, carcasses, and pork food products (fresh meat, fermented and seasoned) from the same pigs were analyzed along the entire food-producing chain. *E. coli* were isolated and tested phenotypically to evaluate their AMR and MDR profiles to five different antimicrobial classes. Similar profiles were then tested using Enterobacterial Repetitive Intragenic Consensus (ERIC-PCR). ERIC is an intragenic repetitive unit based on the amplification of regions between the ERIC sequences that differentiate bacterial strains according to variations in their location. It is adaptable to a wide range of bacterial species, and ERIC sequences were first described in *E. coli* [21]. This technique was used to evaluate the phylogenetic similarities and the potential involvement of food production steps in transmitting antimicrobial-resistant bacteria to consumers.

## 2. Results

### 2.1. Escherichia coli Isolation

Collected samples were processed for *E. coli* isolation. *E. coli* were isolated from all carcass, fresh meat, and fermented meat product samples: 225 strains from carcasses, 62 strains from fresh meat, and 7 strains from fermented meat products. From fecal and seasoned meat product samples, 243/245 and 8/15 *E. coli* were isolated, respectively.

### 2.2. Antimicrobial Resistance Evaluation

**Figure 1:**
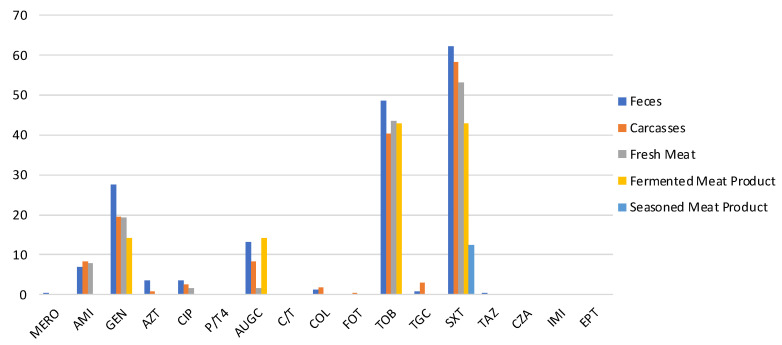
Prevalence of antimicrobial-resistant *E. coli* isolated from feces, carcasses, and meat products. (MERO = meropenem, AUGC= amoxicillin/clavulanic, C/T4 = ceftolozane/tazobactam, FOT = cefotaxime, TAZ = ceftazidime, CZA = ceftazidime/tazobactam, IMI = imipenem, ETP = ertapenem, AZT = aztreonam, AMI = amikacin, GEN = gentamicin, TOB = tobramycin, CIP = ciprofloxacin, COL = colistin, TGC = tigecycline, SXT = sulphamethoxazole/trimethoprim).

All strains were tested for their susceptibility to 17 antimicrobials. The antimicrobial resistances most detected were against SXT, TOB, and GEN, as shown in Figure 1.

In particular, 62.1% (CI 95% = 56–68.2) of *E. coli* isolated from feces, 58.2% (CI 95% = 51.8–64.6) of carcasses strain, and 48% (CI 95% = 36.8–59.2) of pork meat products *E. coli* were resistant to SXT. TOB resistance was observed in 48.6% (CI 95% = 42.3–54.9), 40.4% (CI 95% = 34–46.8), and 39% (CI 95% = 28–50) of *E. coli* isolated from feces, carcasses, and pork meat products, respectively. GEN resistance was detected in 27.6% (CI 95% =22–33.2), 19.5% (CI 95% = 14.3–24.7), and 16.9% (CI 95% = 8.5–25.3) of *E. coli* isolated along the food chain (feces, carcasses, and meat products). From feces, only one strain (0.4%) was non-susceptible to MERO, and one (0.4%) was resistant to TAZ. Moreover, only one strain isolated from carcasses (0.4%) was resistant to FOT. Despite the presence of resistant isolates from feces and carcasses, strains isolated from pork meat products were not resistant to AZT, TGC, or COL (Figure 1). All strains detected along the food chain were susceptible to C/T, P/T4, CZA, IMI, and EPT. 

MDR patterns were evaluated from a farm-to-fork perspective. Particularly, MDR profiles were frequently found in fecal isolates, a few in carcasses, but never in pork meat products. The MDR patterns were: (i) AUGC-TOB-SXT (10/243 fecal *E. coli* and 4/225 carcasses *E. coli*); (ii) GEN-AUGC-TOB-SXT (7/243 fecal *E. coli* and 1/225 carcasses *E. coli*); (iii) AMI-AUGC-TOB-SXT (1/243 fecal *E. coli*); (iv) GEN-CIP-AUGC-TOB-SXT (2/243 fecal *E. coli* and 1/225 carcasses *E. coli*); (v) AMI-GEN-AUGC-TOB-SXT (1/243 fecal *E. coli* and 2/225 carcasses *E. coli*); (vi) AZT-CIP-COL-TOB-TGC-SXT-TAZ (1/243 fecal *E. coli*); (vii) GEN-CIP-TOB-SXT (2/243 fecal *E. coli*); (viii) GEN-CIP-AUGC-TOB-SXT (1/225 carcasses *E. coli*); ix) CIP-TOB-SXT (1/240 fecal *E. coli*); (x) GEN-AUGC-SXT (5/243 fecal *E. coli*); (xi) MERO-AMI-AZT (1/243 fecal *E. coli*); (xii) CIP-AUGC-SXT (1/243 fecal *E. coli*); (xiii) CIP-TGC-SXT (1/225 carcasses *E. coli*).

Moreover, other antimicrobial patterns were evaluated along the food chain (feces, carcasses, meat products). SXT, TOB, CIP, AUGC, SXT-TOB, GEN-TOB, GEN-TOB-SXT, and AMI-GEN-TOB-SXT were considered along the same food chains (Table 1) or in groups of animals that showed the same resistance profile along the food chains evaluated (Table 2). Considering the 12 pork food chains reported in Table 1, phylogenetic analysis was performed on all the *E. coli*.

On farm A, resistance to SXT was detected in feces, fresh meat, and seasoned meat product isolates derived from Pig 3. Moreover, on the same farm, SXT resistance was detected in fecal, carcasses, and fresh meat *E. coli* of Pig 8. On farms C and G, *E. coli* resistant to SXT were found on each farm in carcass samples and their related fresh meat samples (Pig 44 and Pig 57, respectively).

The SXT-TOB resistance pattern was the most frequently found. In fact, on farms B and H, resistant *E. coli* strains were found in the carcasses and fresh meat samples of Pig 34 and Pig 9, respectively (as shown in Table 1). On farm C, one *E. coli* isolated from feces (Pig 27) and one from the carcass (Pig 21) have the same SXT-TOB pattern as their fresh meat isolates (Pig 27 and 21, respectively). On farm G, 2 fecal *E. coli* strains (Pig 51 and Pig 52) showed the same AMR pattern of their meat products (fresh meat and fermented product), and in Pig 55, SXT-TOB resistant strains were found along all the food chain. The AMI-GEN-TOB-SXT pattern was found in *E. coli* isolated from feces and fresh meat belonging to Pig 46 on farm F (Table 1).

As reported in Table 2, 15 groups of pigs were selected for their identical antimicrobial-resistant profile along the food chain. SXT-resistant strains belonging to the same group were identified on farm B. Resistance against TOB was detected in only one group of isolates from farm H (Table 2). CIP and AUGC resistances were detected in carcasses and fresh meat *E. coli* only on farm D—groups 3 and 4.

Three different groups were considered on farms A (group 3), D (group 1), and E (group 1) for their SXT-TOB resistance along the food chain (Table 2). The GEN-TOB pattern was found only in one group of samples belonging to farm C, while the GEN-TOB-SXT pattern was detected in *E. coli* of farms A, B, C, D, G, and H (Table 2). The AMI-GEN-TOB-SXT pattern was found in one group of samples on farm G. 

Other AMR patterns were detected in *E. coli* isolates but were not considered for phylogenetic analysis because they did not harbor the same resistances along the food chain and/or food chain groups. 

Data obtained were compared with information derived from electronic prescriptions collected for the four months before slaughtering, but no antimicrobial treatments were administrated to the pigs selected in that period. 

### 2.3. Phylogenetic Analysis of Antimicrobial Resistant Bacteria

A total of 26 *E. coli* were tested on farm A, 17 isolates on farm B, 16 on farm C, 23 on farm D, 14 on farm E, 2 on farm F, 17 on farm G, and 8 on farm H. Phylogentic analysis performed by ERIC-PCR revealed the presence of 36 clusters.

Analysis of the 12 food chains highlighted the relationship between *E. coli* isolated in Pig 3 (farm A) from feces, fresh meat product, and seasoned meat product in Cluster 6 (C6); in C5 and C32, only feces and carcass *E. coli* (Pig 8 and Pig 55, respectively) were phylogenetically related despite their related fresh meat *E. coli* isolates belonging to another cluster. Feces and fresh meat isolates of the same food chain were related in C14 (Pig 27) and C31 (Pig 51), where they were related to the fermented product, too (Pig 52). Carcasses and fresh meat isolates were related in C15 (Pig 44) and C29 (Pig 57). The other 4 pork food chains were not phylogenetically related (Pig 9, 21, 34). Phylogenetic relations of isolates from Pig 46 (farm F) were not calculated by the software and the UPGMA because the number of considered strains (n° 2) was not coherent with the methodology.

**Figure 2:**
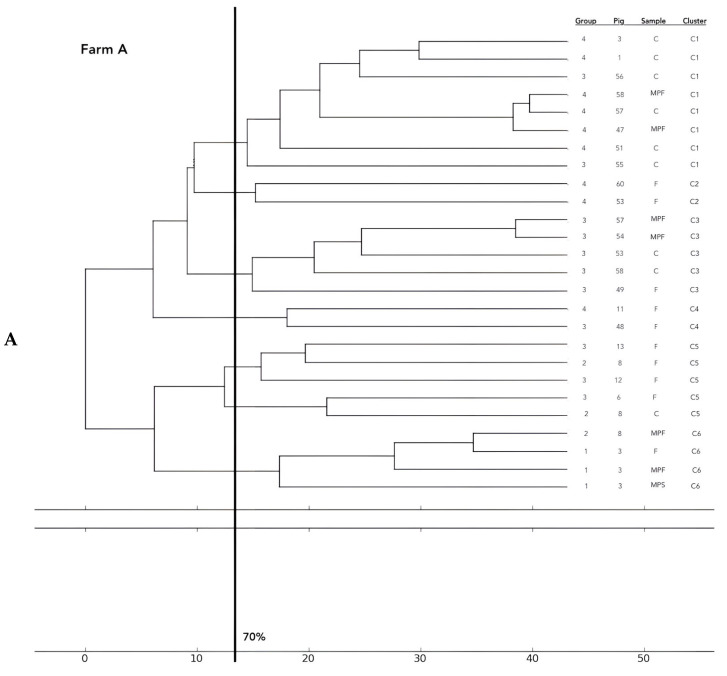

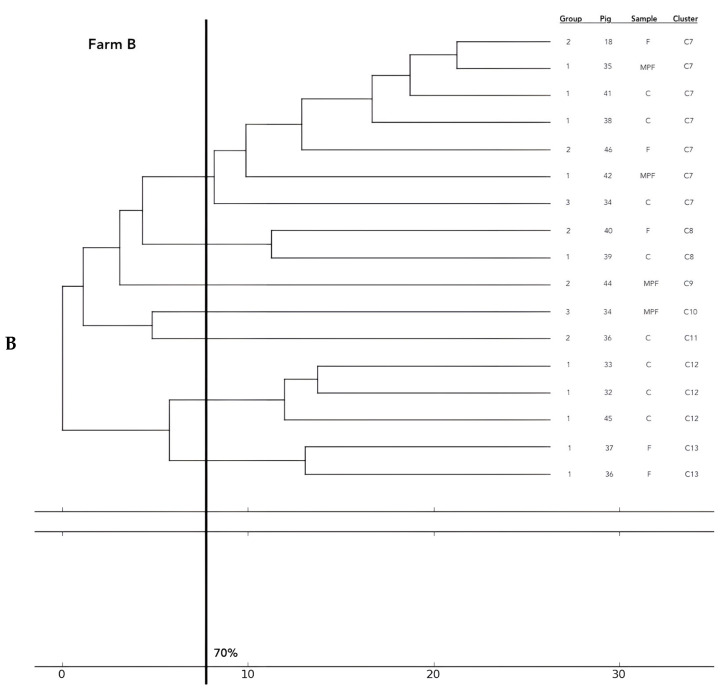

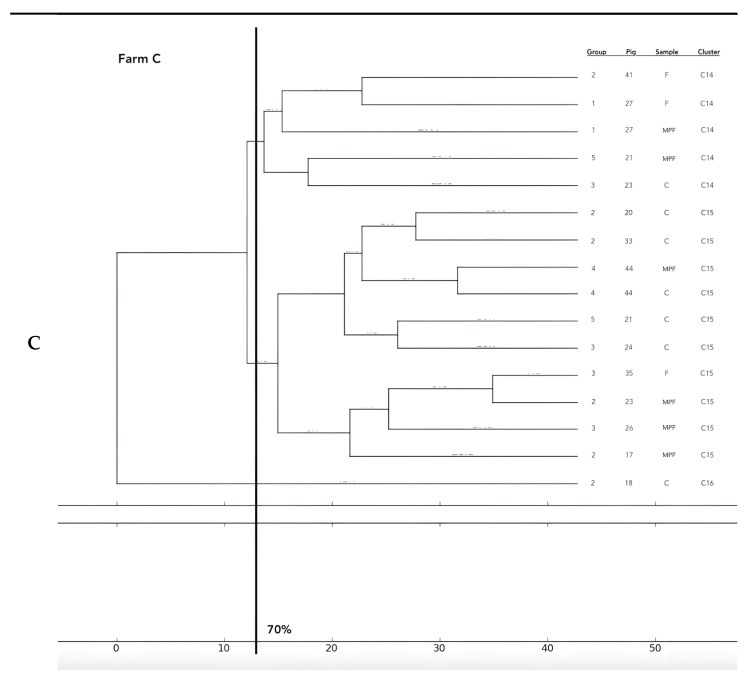

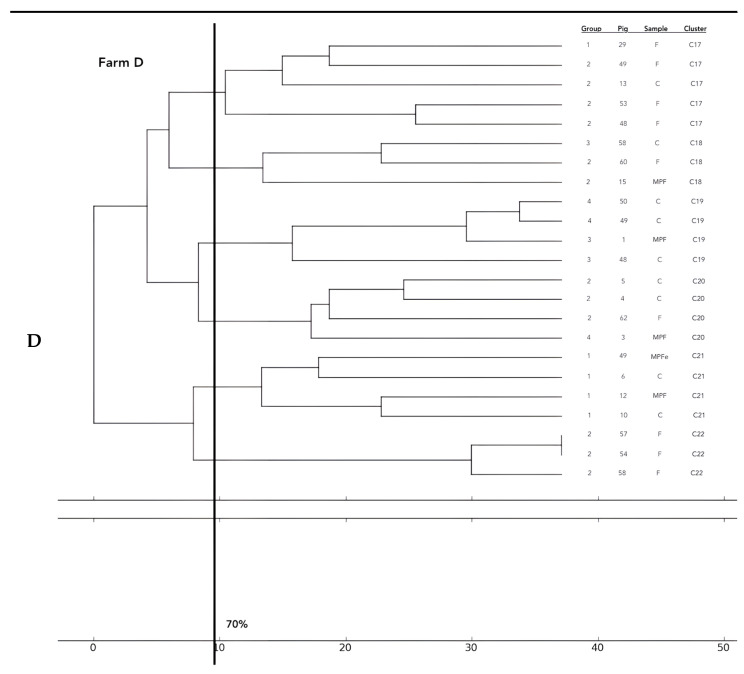

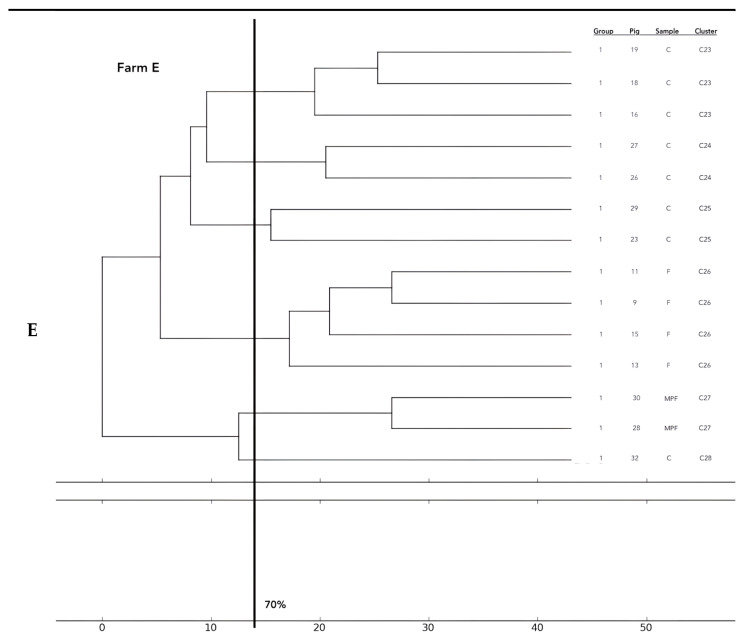

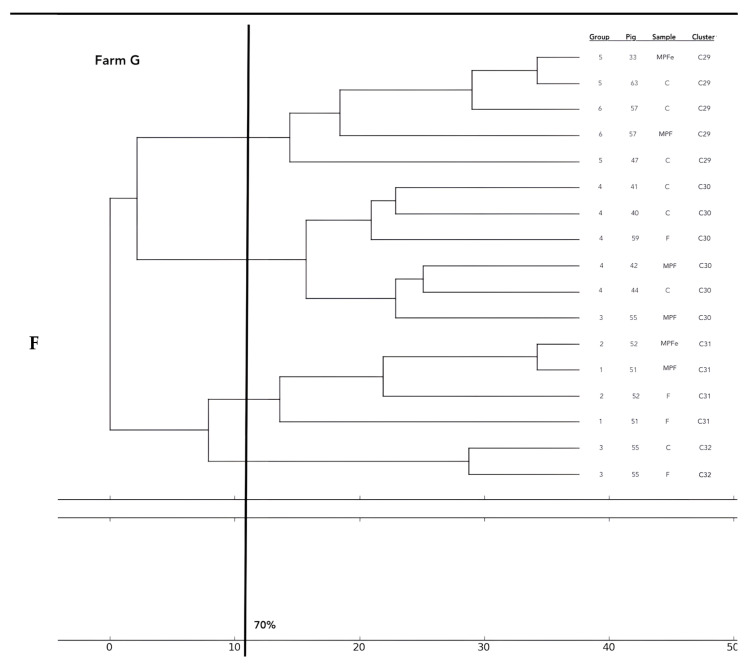

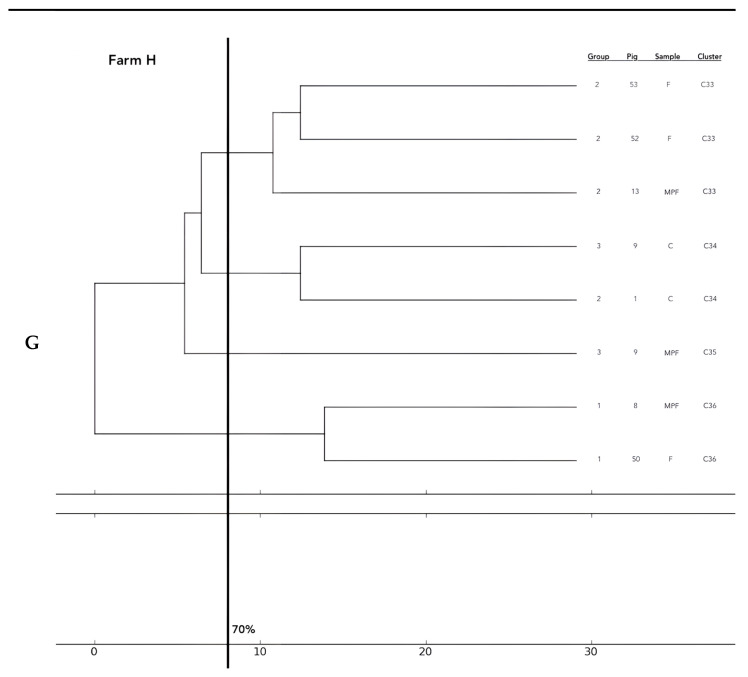
Phylogenetic relations of resistant *E. coli* highlighted in 11 pork food chains and 15 groups of food chain analysis (F = feces, C = carcasses, MPF = fresh meat product, MPFe = fermented meat product, MFS= seasoned meat product). Each subfigure (from (**A**) to (**G**)) represent the phylogenetic *E. coli* similarities in the different pig farms selected.

Considering the phylogentic analysis of 15 groups of pigs along the different food chains, *E. coli* isolated from feces belonging to the same groups harbored phylogenetic similarities in 6/36 clusters (C 2, 5, 7, 13, 22, 26). Moreover, isolates from carcasses of the same groups belonged to the same cluster in 6/36 cases (C1, 12, 19, 23, 24, 25), and *E. coli* of meat products from the same groups showed genetic similarities in only one group (C 27). Frequently, phylogenetic similarities were found in *E. coli* from carcasses and meat products of the same groups (C1, 7, 15, 19, 21, 29) and, in 3/36 cluster (C 3, 15, 30), *E. coli* isolated from feces, carcasses and meat products of the same groups were similar. In 2/36, cluster similarities were found in fecal and carcasses *E. coli* of the same groups (C 17, 20) and in 3/36 in fecal and fresh meat products isolates (C 18, C 33, C 36). All data are reported in Figure 2.

## 3. Discussion

Commensal bacteria in animals are currently recognized as a reservoir of AMR and, at the same time, as a source of AMR transmission. However, the role of longitudinal transmission of those bacteria directly from livestock to humans through meat products is still poorly understood [22]. This study monitored the AMR pattern and phylogenetic relations in *E. coli* isolated from feces, carcasses, and meat products following the pigs along the entire food-producing chain, evaluating the risk of AMR transmission to consumers. According to Commission Implementing Decision 2013/652/EU [23], AMR monitoring in indicator commensal *E. coli* is mandatory in the major domestic producing animal populations and their derived meat. Specific monitoring of extended-spectrum-β-lactamase (ESBL)-, AmpC-, and carbapenemase-producing indicator commensal *E. coli* is also required. According to the EFSA/ECDC 2022 report [24], resistance to ampicillin, sulfamethoxazole, trimethoprim, and tetracycline is high in all European animal categories, frequently causing the development of MDR bacterial profile. Results highlighted in the present study confirm those data, particularly for sulphamethoxazole-resistant *E. coli,* although high levels of resistance to aminoglycosides were also found (GEN and TOB). In fact, in previous studies, aminoglycoside-resistant *E. coli* were frequently detected in pork at slaughter associated with penicillin and tetracycline resistances [25,26]. As reported in other European countries, resistance to colistin, azithromycin, cefotaxime, and ceftazidime is less common, particularly to meropenem. Notably, in this study, only one meropenem-resistant strain isolated from feces was found, while no strain was detected Europe-wide [24]. The MDR *E. coli* pattern is frequently characterized by tetracycline, ampicillin, sulphamethoxazole, and trimethoprim across Europe [24]. In this study, the most frequently highlighted pattern included AUGC-GEN-TOB-SXT and MDR strains detected in feces and carcasses. Fortunately, no MDR strains were isolated from pork meat products. Bacterial isolation along the food production chain in the present study was essential to evaluate the farm-to-fork involvement in disseminating antimicrobial-resistant bacteria. Phylogenetic analysis allowed us to divide strains into 36 clusters and to understand their phylogenetic similarities along the food chain. Data showed that 50% of phenotypical AMR observed along the pork food chain were related phylogenetically. The contamination of fresh meat, in half of the cases, is not directly related to contamination from feces or carcasses. Several studies have reported the transmission from livestock and/or retail meat to humans of ESBL and AmpC β-lactamases plasmid-related genes harbored by *E. coli* strains [26]. Linkages between poultry meat, pork, and humans were detected in the USA. Clones of gentamicin and vancomycin-resistant genes by *Enterococcus* spp. were detected in the feces of healthy humans in Europe and the USA [27,28]. Despite this, some similarities were found in isolates in feces and carcasses. The analysis of groups along the different food chains showed that the most frequent relation was found between *E. coli* isolated from carcasses and meat products of the same groups (16.7%). The frequent relations found between *E. coli* isolated from feces (16.7%) and *E. coli* from carcasses of the same groups (16.7%) highlight possible cross-contamination during farming and processing at slaughter, respectively. In group analysis, phylogenetic similarities from farm-to-fork were detected in the 3/36 cluster (8.3%). Pork can be a reservoir of antimicrobial-resistant bacteria that can be transferred intra and inter-species [25]. This study showed that in the eight selected farms, antimicrobial-resistant bacteria were easily found along the entire food chain, from farm to slaughterhouse to meat products. It is well known that food can be a source of transmission for pathogens. The high demand for ready-to-eat foods and raw or inadequately cooked meals amplifies this phenomenon [29]. To the author’s knowledge, the present study is the first to report a food chain analysis following the same animals directly from farm to meat product. It is necessary to highlight that processed meat products were not frequently found as antimicrobial-resistant *E. coli* carriers. This suggests that, rather than fresh meat, proper seasoning of meat products is still a good method to reduce bacterial load. The study, however, could not evaluate any potential cross-contamination during the handling and processing of food by consumers, which can be an added risk factor [30,31]. At the same time, the contribution of food in the transmission of live bacterial strains (both commensal and pathogen) or resistance genes to humans is still poorly established and underestimated worldwide [14,32]. AMR surveillance in the pig production chain has provided evidence of genetic fingerprint similarities for human nosocomial infection using multi-locus sequence typing (MLST) and whole-genome sequencing [33,34]. In conclusion, this study highlighted the phylogenetic similarities in *E. coli* isolated in all food products, particularly fresh meat. Fresh meat showed a higher risk of AMR transmission than seasoned and fermented meat products. Isolates from seasoned products in this study had a few AMR phenotypic profiles and fewer phylogenetically similarities with isolates obtained from other pork products along the food production chain. Consumers’ awareness of the antimicrobial resistance phenomenon and its possible spread from animals to humans is low. Less than half the consumer population in Europe identified the consumption of meat products as a transfer route of antibiotic-resistant bacteria from animals to humans [35,36,37]. For those reasons, further scientific evidence must be reported.

## 4. Materials and Methods

### 4.1. Sample Collection

Samples were collected from eight different farms (A, B, C, D, E, F, G, H) located in the Emilia Romagna region, North Italy, from 2020 to 2022. Samples included pig feces, pig carcasses, and pork meat products (fresh, seasoned, and fermented meat). Fecal samples were first collected using fecal swabs, and 30 pigs per farm were selected except for farms B and E, where 32 pigs were sampled, and farm H, where 31 pigs were sampled. Each pig was marked with an ear tag and followed along the food production chain. Fecal samples were collected at least 30/40 days before slaughter. The use of antimicrobial treatments on animals was monitored for four months before slaughtering by electronic prescription collection to compare the AMR found and the antimicrobial usage on the selected farms.

The same pigs (30 pigs per farm) were followed to slaughterhouses, and carcass samples were collected using pre-wetted sponges, following ISO 17604:2015 [38] and Reg. CE 2073/05 [39] after the evisceration and half-carcass portioning. Some carcasses could not be sampled for organizational reasons. At the slaughterhouses, fresh meat samples were collected at the end of carcass sampling. Meat products were sampled only after the fecal swab processing and resulting evaluation: pigs that harbored resistant fecal *E. coli* were considered for meat sampling. Only a few of the food chains identified could be sampled for organizational reasons. This procedure was set up so as not to interfere excessively with the production chain. A portion (at least 25 g) was collected as fresh meat, and others were destinated for food transformation. Samples were analyzed at the end of seasoning (coppa, pancetta) and fermentation (salami) processes, which last from 30 to 70 days. The total number of samples collected was 245 fecal swabs, 225 carcass sponges, 62 meat samples, 15 seasoned products, and 7 fermented products. Fermented products did not correspond to one single pig because production foresees using parts of different carcasses together. All the samples were sent to the laboratory of Food Hygiene and Inspection of the Veterinary Science Department, University of Parma.

### 4.2. Escherichia coli Isolation

*E. coli* isolation was characterized by a sample enrichment phase, an isolation phase, and an identification phase following UNI EN ISO 16649-2:2001 [40]. Fecal swabs were enriched via adding 9 mL of Buffered Peptone Water (BPW; Biolife Italiana, Milan, Italy) to the swab in a stile tube and incubated at 37 °C overnight. The enrichment phase of carcass sponges was done by adding the sponge with 225 mL of BPW to a sterile bag and overnight incubation at 37 °C. For fresh meat, seasoned and fermented products, 25 g of representative cross-sections of the samples were incubated at 37 °C overnight in a sterile bag with 225 mL of BPW. The isolation and identification phases were analogous for all the different sample matrices. Using a sterile calibrated handle, the broth culture was streaked onto Triptone Bile X-gluc (TBX; Biolife Italiana, Milan, Italy) agar and incubated at 42 °C overnight. A typical colony was selected and subjected to an indole test. The indole positive colonies were finally confirmed as *E. coli* using the conventional miniaturized API 20E system (bioMérieux, Marcy l’Etoile, France). 

### 4.3. Antimicrobial Resistance Evaluation

All *E. coli* were tested for susceptibility to a set of molecules on Sensititre plates™ (Thermofisher Scientific, Milan, Italy), defining the Minimal Inhibitory Concentration (MIC) following the manufacturer’s instructions. The bacterial suspension inoculated was 5 × 10^5^ CFU/mL, and plates were incubated at 35 ± 1 °C for 18 ± 2 h as defined by European Committee on antimicrobial susceptibility testing (EUCAST), 2020 [41].

Each plate was customized with the following:

- β-lactams: meropenem (MERO: Sensible (S) ≤ 0.25–Resistant (R) > 8), piperacillin/tazobactam (P/T4: S ≤ 8–R > 16), amoxicillin/clavulanic (AUGC: S < 8–R > 8), ceftolozane/tazobactam (C/T4: S ≤ 1–R > 1), cefotaxime (FOT: S < 1–R > 2), ceftazidime (TAZ: S < 1–R > 4), ceftazidime/tazobactam (CZA: S ≤ 8–R > 8), imipenem (IMI: S≤ 2–R > 8), ertapenem (ETP: S≤ 0.5–R > 1); aztreonam (AZT: S ≤ 1–R > 4);
- aminoglycosides: amikacin (AMI: S ≤ 8–R > 16), gentamicin (GEN: S ≤ 2–R > 4), tobramycin (TOB: S ≤ 2–R > 4);
- quinolones: ciprofloxacin (CIP: S ≤ 0.25–R > 0.5);
- polymixin: colistin (COL: S ≤ 2–R > 2);
- glycylcyclines: tigecycline (TGC: S ≤ 1–R > 2);
- sulphonamides: sulphamethoxazole/trimethoprim (SXT: S ≤ 2–R > 4).

The optical density of bacterial growth was recorded at 620 nm by Multiskan FC Version 1.00.75 by Thermofisher Scientific. Resistance to three or more antimicrobial classes defines Multidrug Resistance (MDR) bacterial profile [4].

### 4.4. Phylogenetic Analysis of Antimicrobial Resistant Bacteria

Bacteria belonging to each food production chain were clustered by their AMR pattern considering their sample origin. All the strains that showed the same AMR in feces, carcasses, and meat products of the same pig or the animals originating from the same farm were considered for performing phylogenetic analysis to evaluate whether antimicrobial-resistant bacteria can be transmitted from farm-to-fork. Determination of the *E. coli* isolates’ phylogenetic relatedness was performed using Enterobacterial Repetitive Intergenic Consensus (ERIC-PCR) as described by Ventura et al. 2003 [42]. DNA extraction involved heating at 95 °C for 10 min. The amplification was carried out with a GoTaq G2 Flexi DNA Polymerase kit (Promega Italia S.r.l., Milan, Italy). The master mix was prepared for 25 μL of final volume reaction containing 5x Green GoTaq Flexi Buffer at a final concentration of 1×, 3 mM of MgCl_2_, 0.2 mM of dNTPs, and 2.5 U of GoTaq G2 Flexi DNA Polymerase. Primers ERIC-1 primer (5′-ATGTAAGCTCCTGGGGATTCAC-3′) and ERIC-2 (5′-AAGTAAGTGACTGGGGTGAGCG-3′) [42] were added at a final concentration of 1 μM. A total of 3 μL of sample lysate was added to the reaction mixture and Nuclease Free Water to reach final volume. The PCR protocol and PCR evaluation product are reported by Ventura et al., 2003 [42]. Images were analyzed using Pyelph software 1.4 (Python Software Foundation, Wilmington, DE, USA) through the Unweighted Pair Group Method with Arithmetic Mean (UPGMA) to draw phylogenetic trees from a distance matrix using arithmetic averages of the measure of dissimilarity. Genotyping data were evaluated based on a 70% similarity threshold.

## Figures and Tables

**Table 1:** *E. coli* AMR pattern isolated in the entire food chain (feces, carcasses, and pork meat products of the same pig). The enumeration of “Pig” is independent and related to each farm.

Farm	Resistance Pattern	Feces	Carcasses	Fresh Meat	Fermented Meat Product	Seasoned Meat Product
A	SXT	Pig 3		Pig 3		Pig 3
		Pig 8	Pig 8	Pig 8		
B	SXT-TOB		Pig 34	Pig 34		
C	SXT		Pig 44	Pig 44		
	SXT-TOB	Pig 27		Pig 27		
			Pig 21	Pig 21		
F	AMI-GEN TOB-SXT	Pig 46		Pig 46		
G	SXT		Pig 57	Pig 57		
	SXT-TOB	Pig 51		Pig 51		
		Pig 52			Pig 52	
		Pig 55	Pig 55	Pig 55		
H	SXT-TOB		Pig 9	Pig 9		

**Table 2:** *E. coli* AMR pattern detected in groups of pigs along the different food chains. The enumeration of “Pig” is independent and related to each farm.

Farm	Resistance Pattern	Group	Feces	Carcasses	Fresh Meat	Fermented Meat Product	Seasoned Meat Product
A	SXT-TOB	3	Pig 6, 12, 13, 48, 49	Pig 53, 55, 56, 58	Pig 54, 57		
	GEN-TOB-SXT	4	Pig 11, 53, 60	Pig 1, 3, 51, 57	Pig 47, 58		
B	SXT	1	Pig 36, 37	Pig 32, 33, 38, 39, 41, 45	Pig 35, 42		
	GEN-TOB-SXT	2	Pig 18, 40, 46	Pig 36	Pig 44		
C	GEN-TOB-SXT	2	Pig 41	Pig 33, 18, 20	Pig 17, 23		
	GEN-TOB	3	Pig 35	Pig 23, 24	Pig 26		
D	SXT-TOB	1	Pig 29	Pig 6, 10	Pig 12	Pig 49	
	GEN-TOB-SXT	2	Pig 48, 49, 53, 54, 57, 58, 60, 62	Pig 4, 5, 13	Pig 15		
	AUGC	3		Pig 48, 58	Pig 1		
	CIP	4		Pig 49, 50	Pig 3		
E	SXT-TOB	1	Pig 9, 11, 13, 15	Pig 16, 18, 19, 23, 26, 27, 29, 32	Pig 28, 30		
G	AMI-GEN-TOB-SXT	4	Pig 59	Pig 40, 41, 44	Pig 42		
	GEN-TOB-SXT	5		Pig 47, 63		Pig 33	
H	GEN-TOB-SXT	1	Pig 50		Pig 8		
	TOB	2	Pig 52, 53	Pig 1	Pig 13		

## Data Availability

Data are reported within the article.
